# New Advanced Technologies to Provide Decentralised and Secure Access to Medical Records: Case Studies in Oncology

**DOI:** 10.4137/cin.s965

**Published:** 2009-08-07

**Authors:** Catherine Quantin, Gouenou Coatrieux, François André Allaert, Maniane Fassa, Karima Bourquard, Jean-Yves Boire, Paul de Vlieger, Lydia Maigne, Vincent Breton

**Affiliations:** 1 Inserm, U866, Dijon, F-21000, France; Univ de Bourgogne, Dijon, F-21000, France; CHRU Dijon, Dpt. of Biostatistics and Medical Informatics, Dijon, F-21000, France; 2 Institut Telecom; Telecom Bretagne; Unite INSERM 650 Latim, Brest; 3 Ceren Esc Dijon and Department of Public Health University of Liege; 4 GMSIH, Paris, France; 5 ERIM-ERI 14 INSERM, Faculté de Médecine, Clermont Ferrand; 6 LPC, UMR CNRS-IN2P3 Université Blaise Pascal, Clermont Ferrand

**Keywords:** data security, electronic signature, direct access, medical record, patient identifier, watermarking, grid

## Abstract

The main problem for health professionals and patients in accessing information is that this information is very often distributed over many medical records and locations. This problem is particularly acute in cancerology because patients may be treated for many years and undergo a variety of examinations. Recent advances in technology make it feasible to gain access to medical records anywhere and anytime, allowing the physician or the patient to gather information from an “ephemeral electronic patient record”. However, this easy access to data is accompanied by the requirement for improved security (confidentiality, traceability, integrity, ...) and this issue needs to be addressed. In this paper we propose and discuss a decentralised approach based on recent advances in information sharing and protection: Grid technologies and watermarking methodologies. The potential impact of these technologies for oncology is illustrated by the examples of two experimental cases: a cancer surveillance network and a radiotherapy treatment plan. It is expected that the proposed approach will constitute the basis of a future secure “google-like” access to medical records.

## 1. Introduction

Throughout Europe, patients are entitled to have direct access to their medical records. For example, in France, this has been true since 2002; previously, only indirect access via a physician was allowed. At present, the simplest solution consists in giving to the patient a paper copy of his/her medical record or, if it has been computerized and if the function is available, a digital copy on a readable storage medium. This communication process can be carried out “without constraint at reasonable intervals and without excessive delay or expense” as required by article 12 of the Directive “On the Protection of Individuals with regard to the Processing of Personal Data and on the Free Movement of Such Data”.^1^ Such a delay is needed, however, to ensure that the identity of the individual making the request can be properly checked and that any additional conditions on access, such as those provided for in article 13 section 1(g) of the same directive^1^ “for the protection of the Data Subject or the rights and freedoms of others”, are correctly fulfilled. This current approach does not involve any particular risk to the information system, but there are already pressing demands from patients with their increasingly powerful computing facilities to speed up these processes and to have direct access to medical record systems. These pressures will be difficult to resist in the present, fast moving, electronic environment, and it is difficult to imagine that the traditional, delayed, process will be accepted for much longer. Soon, patients will be expecting to have direct access to their medical files via the internet or its equivalent. Instead of trying to resist this inescapable evolution, it is preferable to seek solutions that provide security for both patients and health professionals while allowing this valuable development in the area of personal freedom and human rights.

However, one on the main problems of direct access is that patient’s medical records, especially in oncology (as several health practitioners of different specialties have to participate in patient care), may be split into different parts and recorded in the information system of different healthcare centres. It is not reasonable to expect patients suffering from cancer to deal with the dispersion of their medical information themselves; they should be provided with a secure way to consult it.

For more than 20 years, Research and Development teams have been working on standardised, centralised, secure and reliable Medical Record (MR) systems. The French DMP project to implement personal MRs for each patient that are accessible to the patient is an illustrative example.^2^ The DMP has raised many difficulties regarding ethical and legal aspects, the definition of a common identifier and the creation of a central storage system for all records. As far as we are aware no country has managed to reach these goals at a national level. An alternative is to develop a strategy based on a pragmatic, secure, distributed, unstructured MR system which could be operational in the very short term. This article promotes a non-centralised and non-standardised MR system based on original search and access to distributed medical data like the one that exists in Israel (Clalit HMO and government hospitals), Pittsburgh (Pennsylvania—UPMC) [1] and is being implemented in Brussels (IRIS hospitals)^3^ and Franche Comte, France (EMOSYST).^4^

Grid technology, which has evolved quickly over the last 10 years, has enabled such an MR system. Today, it has reached a level of maturity in the field of distributed computing and data management, which makes it a natural choice to handle distributed medical data. It allows distributed data sources to be brought together, to be queried remotely and on demand, which mobilizes large CPU (Central Processing Unit) resources to analyse them.

In this paper, we discuss opportunities provided by grid technology to enable secure access to medical records through a google-like interface providing professionals and patients with permanent access to their medical information wherever it has been stored. The advantage of grid technology compared with other existing methods is that no movement of data is needed as they are queried where they are produced. We also discuss security issues beyond information access control (e.g. users’ authentication and assessments of users’ rights) and focus on the need to trace distributed data in order to know where they have come from, to know the last user to consult them and to make sure the data have not been damaged or tampered with. For this purpose, watermarking technologies provide a new way to protect data without interfering with the medical practice.

The paper is organized as follows:

– in section 2, we describe a proposal for a secure access to medical records through a “google-like” interface.
– in section 3, we present grid technology in more detail and provide examples of how it is being used currently to address specific medical needs in oncology.
– in section 4, we discuss a number of issues to be addressed to enable the proposed access to medical records.
– section 5 provides a conclusion and some perspectives.

## 2. Proposal for a Secure « Google Like » Access

Today, the main problem for health professionals or patients who want to have full access to medical information, particularly in oncology, is that this information is very often spread over many medical records kept by different health structures or professionals. Therefore, it would be convenient for the patient suffering from cancer, after identification and authentication, to be able to use a medical search engine to gain access to the medical information that has been selected by the medical practitioner (i.e. suitable for viewing by the patient) wherever it has been stored. The patient can also authorize other medical practitioners (for example if they meet for the first time) to consult his/her information.

First, generally speaking, in industrialised countries each health-care structure whatever the type (public or private) has an information system that gathers structured or unstructured computerised medical records. Secondly, information contained in the routine daily MR is sufficient for the needs of health professionals. Thus, the additional work a doctor needs to do to reconstitute a patient’s medical history (MH) is limited even if the patient frequently consults in different places, as is the case in cancerology. Doctors therefore have this extra workload only occasionally. Given the two previous points, and the dangers and complexity of a centralised system, it seems reasonable to us to set up a system that allows each doctor, once the consent of the patient has been obtained, to collect information on that patient from the different health structures. The doctor will then have to synthesise the patient’s MH for his personal use, save it in his personal information system site, and update it regularly. This effort to synthesise MRs will be reduced because one doctor can pass on information about his patients to other doctors in case the patient moves. For example, the General Practitioner could summarize the patient’s MH which could be accessed by his/her colleagues when necessary, and with the patient’s consent.

The main organisational advantage is that it could be operational rapidly because problems of harmonisation will be reduced and information will be more secure. The decentralised management principle supposes that the saved MR will remain in its unmodified form in terms of content and structure in hospitals and clinics, and will remain identifiable by certain elements that exist in all patients’ MRs such as first names, last names and dates of birth, and require no complementary indexation. When patients or doctors want to gain access to medical data that are distributed among the servers of various hospitals or clinics, they have to be connected to an electronic server on which they identify themselves. In the case of access by a doctor, at the first connection, the patient must be present so as to give his/her consent. This first connection will be made using the doctor’s professional card with a password. In the future, authentication could be ensured by using a professional identity or national identity card based on cryptographic methods. The system would transform the patient’s identity using a cryptographic algorithm. The aim of this algorithm is to obtain a strictly anonymous code, but always the same one for a given individual in order to link all the information concerning the same patient. It would not be possible for the management system to read directly in the memories of the local information system. All of the information would be gathered at the level of the decentralised management system which transfers it to the doctor. The interest of this approach is that it protects the confidentiality of the patient’s identity, particularly during transfer in the network. Only encrypted medical information would be moved. However, to go further about data security, questions must be answered on how to verify that information is reliable and on how to trace data after several copies have been made or when the data come from outside the system. Data reliability relies on proof of information integrity, of its origins and that it belongs to one patient. Though most standards provide for such proof for one transmission, continuity of protection through several transactions is not guaranteed. Hackers who disrupt the confidentiality chain have to be identified and prosecuted.

## 3. Grid Technology for Distributed Medical Data Management

Providing patients with “google-like” secure access to their medical records requires the information to be available for querying and retrieval. Google is able to query and search for any data published on the Internet. However, it will be absolutely necessary to ensure the security of this Internet environment before storing any medical data on it. An alternative is provided by grid technology which allows distributed data to be queried securely according to personal access rights. Grids are defined as fully distributed, dynamically reconfigurable, scalable and autonomous infrastructures to provide location independent, pervasive, reliable, secure and efficient access to a coordinated set of services encapsulating and virtualising resource. Their relevance for managing medical information has been investigated within the framework of the HealthGrid initiative.^5^–^8^ Some platforms in medical data management,^9^ management of paediatric data^10^ or medical radiography data^11^ already benefit from grid technologies to manage medical data securely thanks to dedicated grid middleware services such as MDM^8^ or Globus Medicus.^9^ The use of grids overcomes the difficulties inherent in a centralized storage system, especially high cost and complexity. Grids also make it possible to store data where or very close to where they are produced. Through grid authentication, authorization and accounting, only duly authorized persons can gain access to data which are encrypted and made anonymous when they are transmitted.^12^

Well-identified areas of relevance of the grid paradigm are epidemiology and computer-intensive analysis of geographically distributed medical images. Epidemiology focused on population-level research requires access to distributed, critically sensitive and heterogeneous data, resulting in overall costly computing processes. Users ought to be able to take it for granted that the security mechanisms are sufficient to protect their data; that the results of their research will be private and available to third parties only if designated; that the system will meet the concerns of the ethical and legal committees of their research institutions; that the services are reliable, efficient and permanent; that they do not have to change significantly their current procedures; protocols or workflow, and finally that the data is somehow automatically organised and gathered, and thus available for further exploitation. Early attempts at epidemiological applications of grids^13^ have demonstrated their relevance for patient customized research. In the next chapter, we will present an epidemiological application of grids for cancer surveillance which is currently being used in France.

Another attractive field of application for grid technology is computer-intensive analysis of distributed medical images. The impact of grid technology comes from the secure management of distributed images together with the capacity to gain access to large computing resources on demand to analyze them. In the field of oncology, the use of Computer-Aided Detection (CAD) for the analysis of mammograms was addressed by the MammoGrid project as early as 2005.^11^ Other efforts focus on using grid computing resources to plan radiotherapy treatment:^14^ a case of the use of this technology currently exploited in collaboration with a French Cancer Treatment Centre will be further documented in chapter 4.

### 3.1. Case study 1: cancer surveillance network

Cancer screening programs aim at the early detection of the malignant tumors in order to improve the prognosis. Most EU countries have launched a national program for breast cancer screening.^15^ In France, when a woman is positively diagnosed with a risk of tumour, cancer associations are responsible for providing a second diagnosis on the mammograms and have to follow-up the pathology data about the tumour, which are stored by the laboratories. At present, the patient’s data are faxed on request or carried physically by the patient to the associations where they are recorded again. This process is costly and error prone as data has to be typed and reinterpreted twice.

The cytopathology data are also relevant for epidemiological analysis. The INVS (Sanitary Surveillance Institute), the French equivalent of the (E)CDC in the USA, is in charge of publishing indicators about global health and particularly about cancer. To produce its indicators, the INVS relies on regional cancer registries (CRISAPs) set up to collect relevant information to support statistical and epidemiological studies about cancer incidence, mortality, prevalence or screening. CRISAPs (Centre de Regroupement Informatique et Statistique en Anatomie et cytologie Pathologiques) are like regional data warehouses collecting anonymous data from pathology laboratories or from healthcare establishments involved in cancer treatment. Healthcare professionals in laboratories are reluctant to release data because of cost and also because they lose some control over the data they have produced.

**Figure 1:**
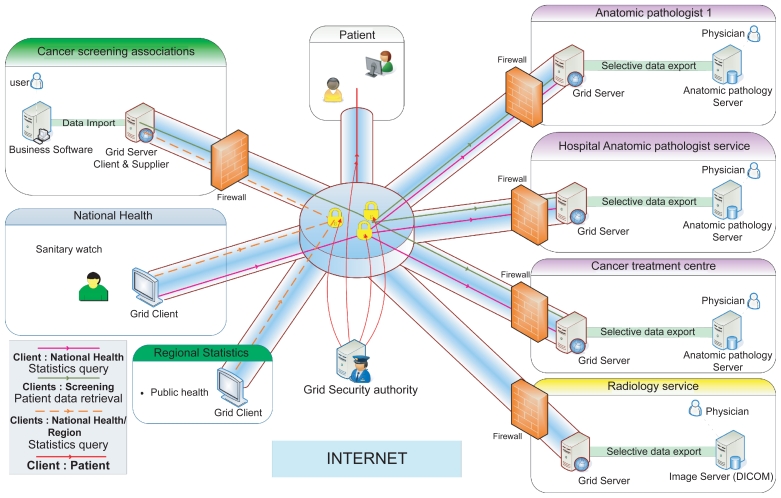
Cancer surveillance network architecture.

An alternative is for clients to query databases of the pathology laboratories. A grid, federating the laboratories, (see Fig. 1) would provide a secure framework enabling the screening associations to query databases and fill their local patient files.^16^ No action is required by physicians to put their data on the network. Thanks to the grid security architecture, the cytopathologists are able to define and modify the access rights of the users querying their data.

Several projects in Europe have studied or are currently exploring the advantages of grid technology with regard to breast cancer, particularly computer-aided diagnosis of mammograms (e-Diamond^17^ and MammoGrid projects).^11^

If a sentinel network is able to federate pathology databases, it can be used by the epidemiological services of the National Institute for Health Surveillance (Institut National de Veille Sanitaire) and the regional epidemiological observatory.

In the present case, it means that women could consult their own data in the pathology laboratories as well as see mammographic images stored in the radiology services through the proposed network.

A cancer surveillance network is presently being implemented in the Auvergne region in France within the framework of the AuverGrid regional grid initiative (http://www.auvergrid.fr) using grid technology developed by the EGEE^18^ (AMGA metadata catalogue^19^ and MDM Medical Data Manager^8^ and Health-e-Child projects^20^ (Pandora Gateway).

### 3.2. Case study 2: application to radiotherapy

Radiotherapy is one of the three major treatments for cancer. It has demonstrated its efficacy in curing cancer and is also the most cost effective strategy. From a technology point of view, radiotherapy is a highly complex procedure, involving many computational operations for data gathering, processing and control. The treatment process requires large amounts of data from different sources that vary in nature (physics, mathematics, biostatistics, biology and medicine), which makes it an ideal candidate for healthgrid applications.

Nowadays, in radiotherapy and brachytherapy, commercial treatment planning systems (TPS), use an analytical calculation to determine dose distributions near the tumor and organs at risk. Such codes are very fast (execution time below one minute to give the dose distribution of a treatment), which makes them suitable for use in medical centres. For some specific treatments using very thin pencil beams (IMRT) and/or in the presence of heterogeneous tissues such as the air-tissue, lung-tissue and bone-tissue interfaces, it appears that Monte Carlo simulations are the best way to compute complex cancer treatment by keeping errors in the dose calculation below 2%. The accuracy of Monte Carlo (MC) dose computation is excellent, provided that the computing power is sufficient to allow for extreme reduction of statistical noise. In order to finish MC computations within an acceptable time period for interactive use, parallel computing over very many CPUs has to be available. In this way, MC dose computations could become standard for radiotherapy quality assurance, planning and plan optimisation years before individual departments could afford local investment that is able to support MC.

With the objective of making Monte Carlo dose computations the standard method for radiotherapy quality assurance, planning and plan optimisation, we are participating in the development of a Monte Carlo platform dedicated to SPECT, TEP, radiotherapy and brachytherapy simulations together with 21 other research laboratories which are involved in the international collaboration OpenGATE (http://www.opengatecollaboration.org).^21^ This GATE software with its accuracy and flexibility was made available to the public in 2004 and now has a community of over 1000 users worldwide. The limiting issue of GATE right now is its time consuming simulations for modelling realistic scans or treatment planning.

A secured web platform enabling medical physicists and physicians to use grid technology to compute treatment planning using GATE Monte Carlo simulations and share medical data has been developed. This platform, named HOPE (Hospital Platform for E-health),^22^ provides quick, secure and easy to use tools to physicians or medical physicists to perform treatment planning on the Grid infrastructure. When the user is logged in, he/she has the possibility to upload or access medical data located on the hospital’s PACS (Picture Archiving and Communication System) server In the case of medical imaging for radiotherapy, the metadata server (AMGA)^19^ services located at the hospital collect metadata as attributes like the name of the patient, the characteristics of the disease, etc. SSL (Secure Socket Layer) connections in addition to encryption systems are used for the transfer of data. Authentication using ACLs (Access Control Lists) are used for the access to metadata in the database. The metadata server provides a replication layer which makes databases locally available to user jobs and replicates the changes between the different participating databases.

Information contained in electronic patient sheets is also registered as parameters in the metadata server. The anonymized medical images are registered on the grid. GridFTP (File Transfer Protocol) is used to enable advanced security transfers. Medical images are associated with patient sheets and the user can automatically visualize them.

By visualizing the tumour, the physician can choose what kind of device is the most appropriate to treat the patient using ionizing particles (field size, type of particle, energy, brachytherapy sources, ...). The treatment plans can be directly visualized from the HOPE portal and downloaded onto the personal computer of the user.

The web portal offers to the user a transparent and secure way to create, submit and manage GATE simulations using realistic scans in a grid environment. The conviviality of the web portal and the Grid performances could make it possible, in the near future, to use Monte Carlo simulations from clinical centres or hospitals to treat patients in routine clinical practice for specific radiotherapy treatments. In addition, the web platform functionalities enable direct access to medical data (patient sheets, images...) and secure sharing between two users located in different hospitals.

## 4. Conditions of Implementation

In the previous section, we have shown how grid technology could provide the services needed to handle and analyze distributed medical data and images securely. In this section, we discuss additional issues that need to be addressed in order to implement the proposed “Google Like” access to medical records.

### 4.1. The implication of assembling medical records in a grid environment: towards an Ephemeral Electronic Health Record (E-EHR)

Storing all heath information in one place was the dream of centralisation proponents, who were certain that it was the only way to deal with the complexity. However, for many years, the public authorities have understood the danger of a centralised system, notably the considerable risk of losing all of the data if the centralised organisation is destroyed. After realising the weaknesses of a centralised system, the USA ministry of defence created in 1969 the ARPANET, a network system that would continue to function in the case of a catastrophe. Considering health data, hackers may see a centralised system as a challenge and try to gain access to the central patient MR system and modify patients’ medical information. Moreover a centralised information system may discourage doctors from providing health information; they may feel less responsible for the information than they do when they hold the information themselves. It may also be difficult for a centralised system to manage and store the huge quantity of information generated. Moreover, any breakdown, however short it may be, would have considerable repercussions given the number of people managed. It would be then safer to store information in different places to ensure protection and privacy.

As a consequence, our proposal for a secure system that allows the patient to gather the different parts of his medical record which may be spread in different healthcare systems may provide an interesting « alternative » to the classical proposal of a centralized medical record. With such a system, during the consultation, the oncologist will have the complete history of the patient thanks to the documents that the medical practitioner has declared as communicable and validated.

It is important to underline the fact that in such a system the medical information of the patients stays in the different hospital or practitioner information systems under the responsibility of the hospital or of the practitioner. This provides better guarantees against attacks than does a centralized system. Moreover, information will be provided to other medical practitioners directly by the patients by transferring the right of access to the practitioner.

### 4.2. Authentication of patients and health professionals before access to medical records on line

Direct access to medical files via electronic media gives rise to many difficulties, and very strict access control and authentication measures are therefore essential, particularly in oncology. The principal difficulty in this field is to ensure that only the holder of the access rights will be able to gain access to the Personal Data.

A brief consideration of the risks associated with unlawful access to Medical Record systems for patients and for healthcare organizations makes it clear that a very reliable authentication system will be required before allowing any public access to such systems. The traditional approach for the authentication of individuals has two components: assertion of identity, followed by proof of the identity.^1^ Generally, this proof can be in terms of something that the individual knows or something that the individual has or something that the individual is. Technical solutions are available to cover any degree of proof in authenticating individuals, but many of them would require a substantial organization system to be set up before they could become effective.

Biometric technologies are sometimes proposed as a way to associate a patient with his or her medical data, as they do not require the patient to bring any documents or remember any information. Though this technology represents real progress both in the identification and in the authentication of the patient, there are still many questions^4^ regarding the accuracy and reliability of each biometric technology and the associated costs. But the main problem lies in the acceptability of such systems by organizations concerned with ethical considerations such as patients’ associations, national ethics committees, human rights associations, and national committees for data protection. For example, in France, the use of biometric solutions for identification in the field of health has not been approved by the National Ethics Committee.

Even today, after extensive computerization of Medical Record systems, the simplest and most common authentication mechanism is still that of an “Identifier” together with a “Password”. This approach combines simplicity of use and management, but it is the weakest and the most unsatisfactory mechanism.^23^ The most satisfactory approach would lie in the creation of an individual chip card including electronic signature cryptographic algorithms,^24^ both for patients and health professionals. Generated signatures will have the value of legal proof in front of a court, as they are legally recognised, and would provide access follow up. It must be noticed that the availability of such a chip card will take some time and will generate considerable expenditure before becoming the accepted standard. As this more satisfactory electronic solution cannot be implemented now and everywhere, only inferior less safe solutions can be considered.

A possible solution is a smart card,^25^–^27^ associated with the provision of a secret PIN code with 8 characters, like the one proposed for use in France for the DMP project. This solution would require hospitals to be equipped with powerful firewall-type data-processing devices to filter access. In such a system, patients provide a list of medical practitioners who are authorized to have permanent access to their medical data. The access rights given to the medical practitioners can be erased at any time by the patient (for example, if he/she moves to another town), who can also authorize temporary access for other medical practitioners he/she has to consult.

In emergency cases, when the patient is unable to express his/her will, the easiest solution is to provide access through a specific procedure (breaking the glass procedure), under the responsibility of the medical practitioner in charge of the patient, with immediate notification to an official security supervisor. This partial solution represents a compromise between security rules and the patient’s health care and ensures that collected data are made available when necessary. It is a general principle of penal law to consider that citizens generally act in accordance with social rules and that penalties are imposed as a deterrent and to punish those who break the law.

Medical records transmitted to patients must be electronically signed by the practitioner to be sure that he/she has agreed to this transfer and that no unauthorized modifications have been made, which is essential in oncology, particularly for pathological descriptions of the disease. Here also, the recognition of the legal value of the electronic signature permits controlled electronic transmission of the medical record to the patient. This electronic signature also makes it possible to ensure that any modifications of the medical record, for example, adding new medical information, are made by the medical practitioner. However, such signatures are ancillary data that can be easily removed.

### 4.3. Verifiying data reliability and tracing outsourced data

As stated previously in section 1, questions arise about how to trace information and verify its reliability. For example, once data are decrypted, they are not protected any more; they can be copied exactly, routed away from the initial use, tampered with, and so on.

Recently, watermarking has been proposed for the protection of medical information.^28^ Basically, watermarking is defined as the invisible embedding or insertion of a message in a host document, for example an image, like XML structured data. Watermarking provides an original way to share a document with some ancillary data like protection data or meta-data, in a way that is a priori independent of the information file storage format. For example, with regard to images, security elements are introduced in the signal by imperceptible modification. Watermarked data thus remain attached at the signal level independently of the image file format. It means that embedded data can be recovered after file format conversion; for example, conversion which occurs when data are outsourced from the system or when they have been screen captured.

Most of the work on watermarking for medical images has been related to the need to verify image integrity (embedding a digital signature inf the image) or improve confidentiality,^28^ as it is often considered that embedding information makes it more difficult for unauthorized persons to gain access to this information. Watermarking is complementary to other security mechanisms. It gives access to a kind of communication channel that is transparent to non-compliant systems, as it does not add extra-header information, while compliant systems will be able to read embedded data.

In the considered framework, before an isolated medical document can be accessed or shared it has to be identified. A watermarked authentication code may allow identification of the health professional who consulted the patient data for the purpose of traceability, or the identification of the patient. To go further, if the embedded identity is rendered anonymous,^29^ then it is possible to gain access to and to link information concerning the same patient without knowing his or her identity so as to guarantee both privacy and interoperability. These patient privacy issues may appear during the verification process, which is necessary to reduce the risk of errors when identifying documents in everyday practice or when sending a patient’s Electronic Health Record. For example, the verifier may be able to gain access to patient data without authorization. This method may also provide a solution to the problem of the identification of lost medical documents. However, further research and development are necessary to extend watermarking methodology to text.

### 4.4. Integration profiles and standards

In the last few years, access to medical data in a distributed or centralized model has been the object of several studies which led to the specification of integration profiles.^30^ Several EHRs projects based on these integration profiles are currently in progress. The main profiles that support the EHRs system are the following:

- The integration profile XDS (cross referencing document sharing) which defines a communication infrastructure based on metadata and using structured (based on standards such as HL7 CDAv2 for documents and DICOM for images) or unstructured data (pdf documents);
- PIX (patient identity cross referencing) and PDQ (patient demographic query) profiles for management of the identity of the patient, which defines how to link the identity of the patient when registered in different organizations or domains);
- BPPC profile for patient consent, which defines the rights attached to a document
- And other security profiles such as ATNA profiles for security of nodes and audit trails.

### 4.5. *Technology against ethics* and *law: the limits of liability*

Even though watermarking methodology allows us to solve the most important part of the problem concerning secure access of the patient to his or her medical record by embedding a strong identification marker in the document, two main dangers still exist. The first lies in the fact that this process of “automatic” access is not accompanied by any medical explanation and even more importantly, there will be no medical warning about the contents that the patient will read. It is by no means certain that providing patients with routine direct access to their medical records automatically extracted from the database is a very satisfactory solution from a medical point of view. If the medical records contain information (like the diagnosis of the cancer) which may cause serious psychological distress (possibly leading to suicide), the hospital or the medical practitioner could be held responsible from a legal point of view or at least from an ethical or deontological point of view. In oncology, the contents of the medical record need to be carefully reviewed (updated or validated) before being delivered to patients. In other cases, information contained in a medical record may refer to third persons (in cases of hereditary diseases, or in cases of divorce), and providing access to such information may be considered a breach of confidentiality. Once again, the hospital or the practitioner may be held legally responsible. Therefore, even though providing patients with automatic access to their medical records appears to be satisfactory from a technical and data-security point of view, it may not fulfil the quality requirements for the security of healthcare information. No transmission should be allowed without the consent of the medical practitioner who takes care of the patient, or the practitioner’s representative. As the practitioner is legally responsible, his/her formal agreement to the transmission is required, and the transmitted document should be electronically signed by him/her.

The second point lies in the use of the medical record by the patient. As patients are deemed to be responsible adults, we will not consider the possible unexpected effects of communicating their medical records to their insurance company or bank, which may have required it officially or unofficially. From a medical point of view, the main problem could come from modifications of the medical record by patients themselves to erase information that prevents them from obtaining certain advantages. If such modifications were possible, imagine what could happen if patients erased the fact that they had cancer in order to get a job. Thus, it does not seem desirable to give to everybody direct access to the system that manages the files, even to authenticated users. However, it seems reasonable to verify integrity and authenticity of outsourced data, especially when they are used in a healthcare framework. The original medical record, which is the means to bring evidence in case of litigation, should be protected from any kind of attempt by unauthorised persons to modify the information. It will then be preferable to envisage a request procedure for access, including the search for the file and the extraction of the communicable documents authorized by the law. This approach, in which a special access file is created, could happen much faster than the time delay allowed in some European countries (in the UK the authorities have 40 days to comply with a Subject’s Access request, whereas in France, the delay is 8 days).

## 5. Conclusion

The idea that every citizen will have direct access to his medical records anywhere is no longer a utopia, as this situation can be considered the logical outcome of much of the work that is going on world-wide in e-Government, e-Health and e-Shopping. With the constant progress of technology, it is now possible to envisage the possibility of “reassembling” personal health records anywhere and anytime. But security measures need to be much stronger, necessitating mechanisms to provide continuous data protection.

In this paper we have illustrated the potential of grid technology for medical data record sharing through its use in two experimental cases. The deployment of such technology needs to be coupled with relevant security measures and mechanisms. Strict identity checks for both the patient and healthcare professionals are necessary and can be based on cryptographic techniques as those planned for the electronic signature. They can also be used to ensure the confidentiality and integrity of files, authentication of the applicant’s identity and access follow-up. In addition, watermarking technology can provide ultimate protection once access control is bypassed. This would ensure the reliability and traceability of data inside and outside the system. With regard to search engines, who could have imagined ten years ago that a system would be able to retrieve everything you have ever published and list all the people who have made a reference to it in a matter of seconds!

## Figures and Tables

**Figure 2:**
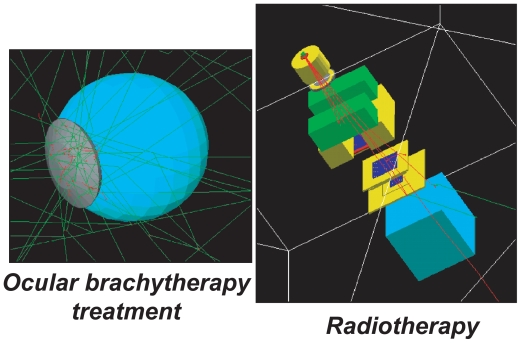
Examples of GATE Monte Carlo simulations for radiotherapy treatment.

**Figure 3:**
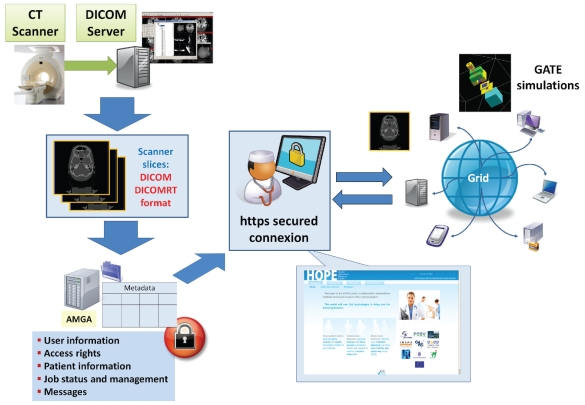
HOPE (Hospital Platform for E-health) web platform.
